# Cultivation and characterisation of *Salicornia europaea*, *Tripolium pannonicum* and *Crithmum maritimum* biomass for green biorefinery applications

**DOI:** 10.1038/s41598-022-24865-4

**Published:** 2022-11-28

**Authors:** Laura S. S. Hulkko, Ariel E. Turcios, Stéphane Kohnen, Tanmay Chaturvedi, Jutta Papenbrock, Mette Hedegaard Thomsen

**Affiliations:** 1 AAU Energy, Aalborg University, Niels Bohrs Vej 8, 6700 Esbjerg, Denmark; 2 Institute of Botany, Leibniz Universität Hannover, Herrenhäuser Straße 2, 30419 Hannover, Germany; 3 CELABOR, Avenue du Parc N°38, 4650 Herve, Belgium

**Keywords:** Chemical engineering, Crop waste, Biofuels, Salt

## Abstract

Salt-tolerant halophytes have shown potential for biorefinery and agricultural use in salt-affected soils, increasing the value of marginal lands. They could provide a bio-based source for compounds obtained from the petrochemical industry or an alternative for biomass currently imported overseas. *Salicornia europaea*, *Tripolium pannonicum* and *Crithmum maritimum* were cultivated in hydroponic systems under various salinity conditions, harvested green but not food-grade, and fractionated to green juice and fibre residue. Obtained fractions were characterised for contents of carbohydrates, Klason lignin, crude protein, organic acids, lipids, and minerals to evaluate the biomass’ suitability for biorefinery. Significant differences were observed in the biomass yield and the composition of the biomass fractions from different cultivation salinities. High concentrations of crude protein were found. Thus, these species could have the potential for green protein production. Fractions rich in carbohydrates could be used for lignocellulose processing and processes utilising micro-organisms.

## Introduction

Soil salinisation has been reported as one of the major factors to the degradation of agricultural land^1^. The Food and Agriculture Organization of the United Nations (FAO) estimates that 79 million hectares are considered either salt-affected or sodic in Europe, covering 3.9% of the total land area^2,3^. Worldwide, salt-affected soils consider more than 100 countries, and total area of saline and sodic soils is estimated to be more than 1 billion hectares^2^. According to The United States Department of Agriculture, 10 million hectares of farmland is lost every year due to over-irrigation and poor water management^4^. Most conventional crops are glycophytes, meaning that their growth is inhibited in the presence of salt, and a 50% decrease in biomass yields have been reported for rice, durum wheat and barley at salinities of 80 mM NaCl, 100 mM NaCl and 120 mM NaCl, respectively^4^. FAO defines saline soils as areas, where the electric conductivity of soil extract is 4 dS/m or higher^2^, which corresponds to approximately 40 mM NaCl. Besides economic losses, soil salinisation is a threat to food security, as it creates challenges to meet the demand for food for the world’s increasing population.

The natural habitat of halophytes are seashores, marshes and salt deserts, and utilisation of these naturally salt-tolerant plants in agricultural applications is a key to re-valorise these salt-affected marginal lands, which are not suitable for conventional farming^3,4^. Flowers and Colmer^5^ define halophytes as plants that can complete their full life cycle and reproduce under the salinity of 200 mM or more, and these type of plants cover approximately 1% of known plant species. Some halophytes can yield as much biomass as traditional crops under full seawater irrigation^6^. Several cultivation practises for saline agriculture has been set for halophytes, including field or greenhouse cultivation with brackish or seawater irrigation, constructed wetlands, or saline hydroponic systems^6,7^. Hence, the irrigation water from valuable freshwater resources may not be needed for cultivation. Due to their capability to grow in hydroponics and constructed wetlands, plants can also be used to bio-filter excessive nutrients^8^ or residues of antimicrobial compounds^9^ from aquaculture effluents. Combining aquaculture with *Salicornia* cultivation has also been evaluated for its potential to reduce halophyte production costs^10^. The most suitable cultivation system depends on the species, salinity of the irrigation water, and available soil type^7^.

Halophytes have previously been used as medicinal plants, and nowadays, fresh tips of many edible species are sold for culinary use^4,11^. After harvesting for food, the remaining fraction of halophyte is often seen as waste as the plant lignifies, trapping high concentrations of salt within the plant structure. Due to this high salt concentration, halophytes are suitable for animal feed only when incorporated with other feed sources. The halophyte-supplemented feed has been tested for aquacultures^12^, chicken^13,14^ and ruminants^15,16^.

According to the IPCC 2021 report^17^, humans as a society have to aim for net-zero carbon dioxide emission by 2050 in order to limit global warming to 1.5 °C. As a part of this green transition, it is necessary to find bio-based alternatives to the variety of products currently obtained from the petrochemical industry. Considering bioenergy, *Salicornia* spp. have been tested for their potential for bioethanol^18,19^ and biodiesel^20,21^ production, and *Tripolium pannonicum* has been tested for biogas production^9,22^. As the production of only biofuels is rarely feasible, value-added products can be introduced to the biorefinery process. These multi-product systems are seen as the most robust option for the future^23,24^. The utilisation of residual fractions for bioenergy production would also lead to a zero-waste biorefinery.

Green biorefinery, where fresh but non-food grade biomass is fractionated to green juice and fibre residue, could provide an opportunity to produce protein-rich feed supplements^25^ and nutraceutical compounds, such as polyunsaturated fatty acids and carotenoids^26,27^. This approach has been tested previously for *Salicornia sinus-persica* and *Salicornia bigelovii*^19,28^. One of the key compounds to be valorised in green biorefinery is protein and halophytes could provide a source for locally produced feed. The demand for high-quality plant-derived protein is increasing and farmers are currently strongly dependent on imported sources, such as soybean, for their livestock^25^. High-value bioactive compounds suitable for cosmetics and pharmaceutical ingredients can also be found from *Salicornia* spp.^27,29–32^, *Tripolium pannonicum*^33,34^ and *Crithmum maritimum*^35,36^ extracts, and these compounds have exhibited antioxidant activity, anti-inflammatory and antimicrobial effects, anti-obesity properties, and even cancer-preventing capabilities.

Halophytes could provide a valuable feedstock for multi-product green biorefinery. Regardless of their potential, halophytes are currently underutilised in agriculture and industrial applications. In this study, three halophyte plants native to European seashores are cultivated and characterised: *S. europaea* (glasswort, marsh samphire, sea asparagus, pickleweed or sea beans), *T. pannonicum* (sea aster, previously defined in taxonomy as *Aster tripolium*) and *C. maritimum* (sea fennel or rock samphire). These species represent different plant families (Amarathaceae, Asteraceae, and Apiaceae, respectively) and they have all suggested as potential crops for halophyte-based agriculture by Ventura et al*.*^4^. The focus is to study the effect of cultivation salinity on biomass yields and the chemical composition of biomass fractions. The plant material was characterised for the contents of carbohydrates, Klason lignin, organic acids, crude protein, lipids, and minerals, for the further evaluation of the species’ suitability for green biorefinery and different types of processes.

## Methods

### Biomass cultivation

The plants were cultivated in a greenhouse at the Institute of Botany, Leibniz University Hannover, Germany (52°23′42″N; 9°42′13″E), with temperatures varying between 14 °C (minimum temperature during the night) and 35 °C (maximum temperature during the day). The seeds of *T. pannonicum* (Jacq.) Dobrocz. were collected with official permission at the North Sea, Germany (53°29′13″N; 8°03′16″E). The formal identification of the species was carried out at the Institute of Botany, Leibniz University Hannover, and the voucher specimen was deposited in the herbarium with specimen number TP20191001. Seeds of *S. europaea* L. var. Aprica were obtained from Serra Maris bvba, Belgium, and the seeds of *C. maritimum* L. were obtained from mother plants grown at the Institute of Botany, Leibniz University Hannover. The agronomic handling from sowing through transplanting was carried out as described by Buhmann et al.^37^. *S. europaea* and *T. pannonicum* were cultivated with different NaCl concentrations in hydroponic systems: 0, 10, 20, 30, and 40 g/l NaCl (corresponding to 0, 171, 342, and 685 mM NaCl, respectively). It was noted that *C. maritimum* did not survive under the highest salinities. Hence, it was grown with lower salinities: 0, 5, 10, 15, and 20 g/l NaCl (corresponding to 0, 86, 171, 257, and 342 mM NaCl, respectively). All plants were cultivated in polypropylene containers (400 mm × 300 mm × 175 mm) with a capacity of 16 l, and each container had 13 l of Hoagland solution containing: 606 mg/l KNO_3_, 944 mg/l Ca(NO_3_)_2_·4H_2_O, 230 mg/l NH_4_H_2_PO_4_, 246 mg/l MgSO_4_·7H_2_O, 3.73 mg/l KCl, 1.55 mg/l H_3_BO_3_, 0.34 mg/l MnSO_4_·H_2_O, 0.58 mg/l ZnSO_4_·7H_2_O, 0.12 mg/l CuSO_4_·5H_2_O, 0.12 mg/l MoNa_2_O_4_·2H_2_O, and 9.16 mg/l Fe-EDDHA (0.56 mg/l Fe). Small compressors constantly aerated the water, and one air stone was placed in the middle of each tank. The hypocotyl was fixed with soft foam in 35 mm holes. The water level was adjusted constantly in each tank with tap water to compensate for evapotranspiration. Each experimental unit consisted of eight plants per container and three replicates (separate containers) per treatment. Plants were exposed to 14 h of artificial light from sodium vapour lamps (SON-T Agro 400, Philips), and the light intensity ranged from 65 to 850 µmol m^−2^ s^−1^ depending on the time of the year, the time of the day, and the weather conditions. The cultivation time in hydroponic systems was 5 weeks for *T. pannonicum* and *S. europaea* and 11 weeks for *C. maritimum*. Plants were harvested partly lignified, weighed, immediately frozen in liquid nitrogen to inhibit metabolism, and then kept at − 80 °C.

### Biomass fractionation and processing

Biomass processing and characterisation were performed in AAU Energy, Aalborg University, Denmark. Harvested aerial parts of biomass were thawed and fractionated to green juice and fibre residue by using a horizontal single-auger juicer. Both fractions were recovered to pre-weighed containers. The contents of dry matter (DM) and ash in green juice and fibre residue fractions were determined using the analytical protocols by National Renewable Energy Laboratory (NREL)^38,39^. The juice was analysed unfiltered and contained small suspended solid particles, which passed through the particle retention of the juicer. For storage, the fibre residue was dried overnight at 60 °C in a fan oven, knife-milled to particle size < 2 mm and kept at room temperature in dry conditions protected from light. Green juice was frozen after fractionation and kept at − 40 °C before composition analysis.

### Characterisation methods

#### Crude protein determination

The crude protein content of the biomass was determined from homogenised DM by measuring the total nitrogen content using an elemental analyser and applying the Jones conversion factor of 6.00^40^.

#### Extraction of lipids

The lipid content was defined as a lipid-enriched non-polar fraction in the biomass. For solid biomasses, a dried sample (3–5 g) was weighed onto a cellulose thimble. Lipids were extracted with 250 ml n-hexane using the Soxhlet apparatus with a 100 ml extraction chamber. After extraction, the solvent was recovered using a rotary evaporator, and extracted non-polar compounds were weighed.

In order to extract non-polar compounds from green juice, liquid–liquid extraction was performed by mixing a juice sample (10 ml) with n-hexane (20 ml) in Falcon tubes. Tubes were kept in a nutation mixer for 1 h at room temperature, and juice solids and liquid phases were separated afterwards by centrifuging for 15 min with 4000 rpm (SL16, Thermo Scientific). The non-polar fraction was recovered, the solvent evaporated in the fume hood, and the lipid-enriched fraction was weighed.

#### Total carbohydrates and organic acids

To analyse the lignocellulosic fraction of solid samples, subsequential 10 h water and 8 h ethanol extractions were performed to remove non-structural compounds. Extraction was performed in a similar setup as used for lipid extraction, and one sample (5 g) was used from each biomass batch. The extracted material was recovered and weighed. Strong acid hydrolysis and determination of structural carbohydrates and Klason lignin in the extractive-free biomass were carried out in duplicate using a protocol by NREL^41^. Carbohydrates from juice fractions were measured after weak acid hydrolysis, where the juice sample (10 g) was mixed with H_2_SO_4_ (10 ml, 8%) in Pyrex tubes and autoclaved at 121 °C for 10 min. Hydrolysis and analytics were run as duplicates for samples and recovery standards. Free sugar monomers and the concentration of organic acids were also determined directly from fresh, untreated juice samples using a protocol by NREL^42^.

All hydrolysates and fresh juice samples were filtered through 0.22 µm syringe filters. Sugars and organic acids were analysed with high-performance liquid chromatography (*1260 Infinity II, Agilent Technologies*), using H_2_SO_4_ mobile phase (0.005 M), organic acid column (*Aminex HPX-87H, Rio-Rad Laboratories Inc.*), and refractive index detector. Separated sugars were glucose, xylose and arabinose, and the calculations for the concentrations of sugars were performed as described by Alassali et al.^19^. Analysed organic acids were lactic acid, acetic acid, malic acid, succinic acid and glycolic acid.

#### Mineral analysis

The minerals present in the ash fraction were analysed at the scientific service centre CELABOR, Belgium. Ash samples were digested in acidic conditions under pressure (40 bar) at 240 °C using a microwave system in compliance with EN 13805:2014 standard. Concentrations of the following minerals were measured using inductively coupled plasma atomic emission spectrometry *(8300 DV ICP-AES, Perkin Elmer*) with a method adapted from EN 11885 standard: aluminium, antimony, arsenic, barium, cadmium, cobalt, chromium, copper, iron, potassium, magnesium, manganese, molybdenum, sodium, nickel, lead, silver, selenium, titanium, zinc, phosphorus, strontium, vanadium, thallium and calcium. Inductively coupled plasma with mass spectrometry (*820 ICP-MS CRI, Varian*) with a method adapted from EN 15763 standard was used to determine the concentrations of rubidium, scandium and yttrium. Detected minerals with a concentration higher than 100 ppm in DM were reported. Analysis was run only once to each ash sample; thus, results are presented in Supplementary Information.

### Statistical methods

All analyses were carried out as triplicates unless stated otherwise. All results, excluding fractionation yields and results from the mineral analysis, are given as mean values with standard deviation. One-way analysis of variance (ANOVA) coupled with Tukey honest significance test was used to test the statistical significance of differences between results from biomass batches cultivated in different salinity conditions.

### Ethics

The study complies with local and national guidelines.

## Results

### Biomass yields

In all plant species, significant differences were observed between biomass yields from different cultivation salinities (*p* < 0.001). The lowest total yield of *S. europaea* biomass (1586 g) was gained from the non-saline cultivation conditions, whereas the highest total biomass yield (4365 g) was achieved with a cultivation salinity of 342 mM NaCl.

**Figure 1:**
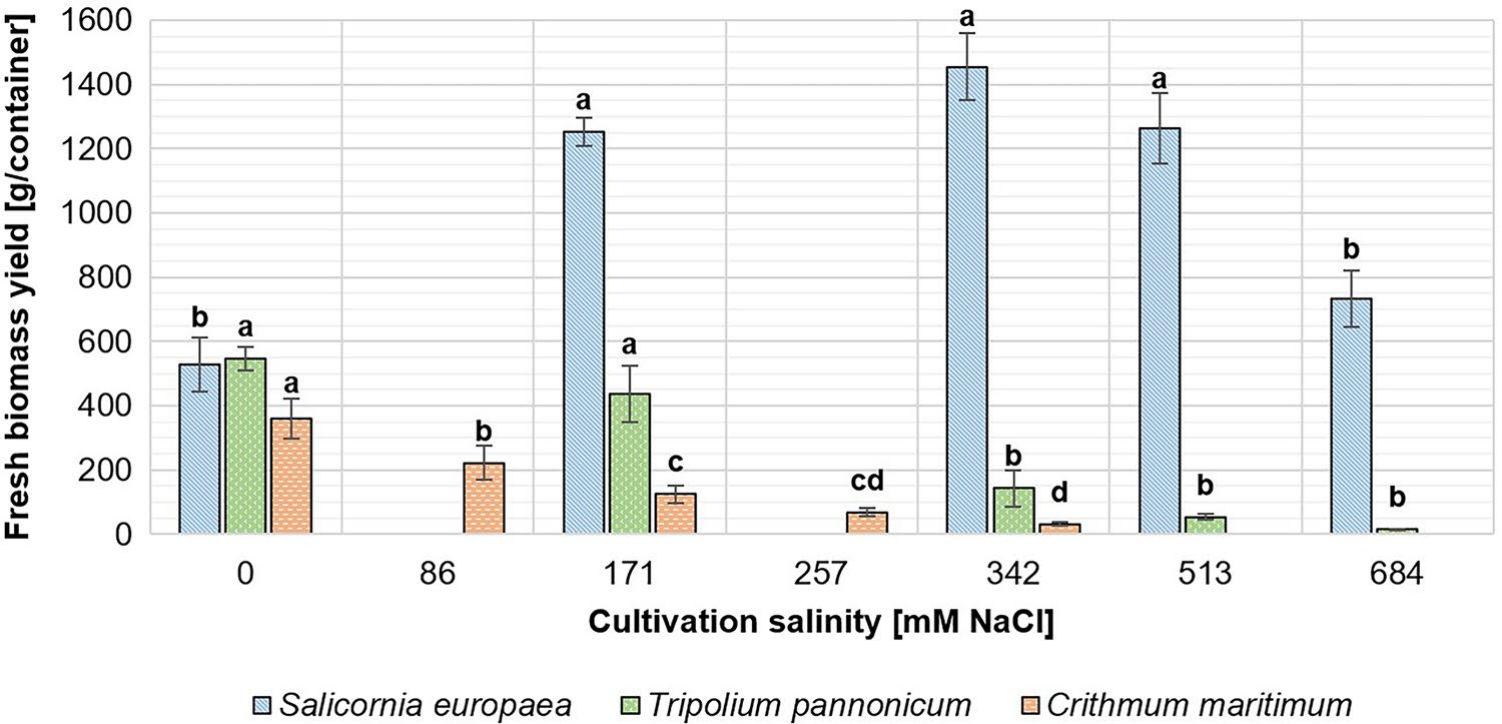
Yield of fresh halophyte biomass cultivated under different salinities. Different letters above the bars stand for significantly different (*p* < 0.05) results calculated individually for each plant species.

On the other hand, there was an inverse relationship between cultivation salinity and *T. pannonicum* yield. The total amounts decreased from 1641 g of fresh biomass (0 mM NaCl) to 44 g (684 mM NaCl). Similarly, the total *C. maritimum* yield decreased from 1440 g (0 mM NaCl) to 126 g (324 mM NaCl). *C. maritimum* also exhibited lower biomass production compared to two other species. Obtained biomass yields are presented in Fig. 1.

### Fractionation and dry matter determination

After fractionation, green juice covered 84.6–90.2 w/w% out of the total biomass. Biomass yields of *T. pannonicum* and *C. maritimum* cultivated in high salinities (> 171 mM NaCl) were too low to perform the fractionation process. Hence, these plants were considered only as whole shrubs. In *S. europaea* and *T. pannonicum*, the juice fraction increased when cultivation salinity increased, whereas, in *C. maritimum,* the juice fraction decreased.

There was a positive correlation between *S. europaea* juice DM content and cultivation salinity, as water-soluble salt is mainly present in the juice fraction. The total DM content of *T. pannonicum* and *C. maritimum* biomasses increased as cultivation salinity increased, and significant changes were observed between samples (*p* < 0.001). In contrast, the changes in the total DM content of *S. europaea* were non-significant (*p* < 0.058). Halophyte fractions and their respective DM contents are summarised in Table 1. The characterisation results for compounds in juice and fibre residue fractions are given on the basis of their respective DM and the total DM calculated from these two fractions (whole biomass).

**Table 1:** Green juice and fibre residue fractions obtained from halophyte biomass and their respective dry matter (DM) contents, and the total DM content of the biomass.

Species	Salinity [mM NaCl]	Juice [w/w%]	Fibres [w/w%]	DM_juice_ [w/w%]	DM_fibres_ [w/w%]	DM_whole_ [w/w%]
*S. europaea*	0	84.61	15.39	4.71 ± 0.03^c^	27.24 ± 1.18^a^	9.15 ± 0.65^a^
	171	85.71	14.28	4.71 ± 0.04^c^	23.00 ± 0.28^b^	n/a
	342	85.29	14.71	5.10 ± 0.00^bc^	21.80 ± 1.16^b^	8.02 ± 0.38^a^
	513	86.42	13.58	5.26 ± 0.39^ab^	21.11 ± 0.54^b^	7.85 ± 0.74^a^
	684	90.23	9.77	5.82 ± 0.12^a^	21.54 ± 1.32^b^	8.05 ± 0.04^a^
*T. pannonicum*	0	74.97	25.03	3.87 ± 0.03^b^	23.78 ± 0.20^b^	8.07 ± 0.19^c^
	171	82.51	17.49	4.17 ± 0.04^a^	26.30 ± 1.23^a^	7.96 ± 0.67^c^
	342	n/a	n/a	n/a	n/a	11.45 ± 0.39^b^
	513	n/a	n/a	n/a	n/a	11.47 ± 0.47^b^
	684	n/a	n/a	n/a	n/a	14.65 ± 0.22^a^
*C. maritimum*	0	70.35	29.65	5.58 ± 0.02^b^	27.22 ± 0.68^a^	11.06 ± 0.22^b^
	86	70.46	29.54	5.39 ± 0.00^c^	27.15 ± 0.35^a^	11.77 ± 0.53^b^
	171	66.97	33.03	7.44 ± 0.00^a^	26.06 ± 0.61^a^	13.56 ± 0.54^a^
	257	n/a	n/a	n/a	n/a	13.40 ± 0.14^a^
	342	n/a	n/a	n/a	n/a	14.79 ± 0.98^a^

Due to small biomass yields, batches of *T. pannonicum* and *C. maritimum* cultivated in high salinity conditions were not fractionated. Results are expressed as [w/w%] on a fresh weight basis. Different letters denote significantly different (p < 0.05) results calculated individually for each plant species and its biomass fractions.

### Composition of halophyte biomasses

#### Salicornia europaea

**Figure 2:**
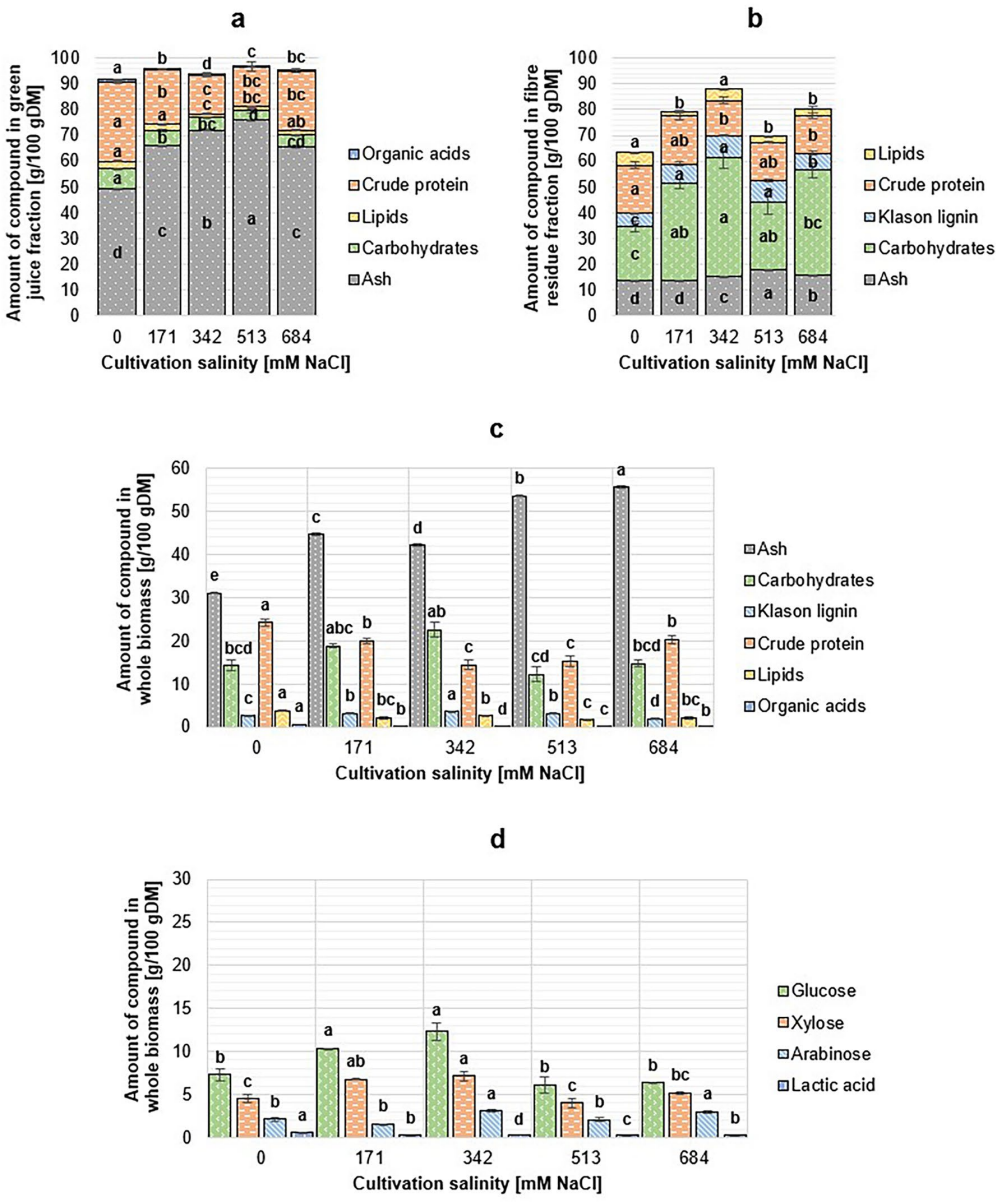
Chemical composition of *Salicornia europaea* green juice (**a**), fibre residue (**b**) and whole biomass (**c**), and total sugar profile (**d**). DM: dry matter. The content of a compound in whole biomass was calculated from juice and fibre residue fractions. Different letters above the bars denote significantly different (*p* < 0.05) results calculated individually for each biochemical group.

The composition of *S. europaea* biomass fractions are shown in Fig. 2. The crude protein content of *S. europaea* was relatively high, and considering the biomass fractions separately, the changes in the crude protein content were more significant in the juice fraction (*p* < 0.001) than in the pulp fraction (*p* < 0.011). Out of the total crude protein, > 60% was present in the juice fractions, except for the *S. europaea* cultivated at 513 mM NaCl, where most of the protein was found in the fibre residue fraction. The lipid content of green *S. europaea* was low, 1.7–3.7 g/100 g DM. Based on the results, no clear relationship between cultivation salinity and the lipid content of the halophyte biomass was observed.

The analysis showed the content of carbohydrates in the biomass being highest when the biomass was cultivated in more optimal conditions in terms of biomass yield (342 mM NaCl). Significant differences were observed for all types of sugars measured in the hydrolysates from juice and fibre residue (*p* < 0.001). Contrary to the total carbohydrate content, in the green juice fractions, the concentration of carbohydrates determined from hydrolysates had an inverse relationship to cultivation salinity, the concentration of sugars being highest in the juice obtained from 0 mM NaCl cultivated plants. Therefore, the carbohydrates from the fibre fraction are more pronounced in the total carbohydrate content of the biomass. Overall, the Klason lignin content was very low in all *S. europaea* fibre residues. Similarly to the total carbohydrates, the lignin content correlated with the obtained biomass yields (more lignocellulose from more optimal cultivation salinity). In *S. europaea* plants, even the highest share of lignocellulose was only 26.1% of the total biomass composition (342 mM NaCl). The average composition of lignocellulose was 43.5% cellulose (glucose), 41.3% hemicellulose (xylose and arabinose) and 15.2% Klason lignin.

In the fresh juice (non-hydrolysed sample), low concentrations of free sugar monomers were detected. The glucose concentration was the highest in the juice from biomass grown in 0 mM NaCl (2.15 ± 0.01 g/100 g DM) and the lowest in the juice from 513 mM NaCl cultivated biomass (0.91 ± 0.79 g/100 g DM). Arabinose was not present in any fresh juice samples, and xylose was detected only from juice samples from 171 mM NaCl (2.06 ± 0.02 g/100 g DM) and 684 mM NaCl (1.29 ± 0.00 g/100 g DM) cultivated biomass. The results of the organic acid analysis showed only low amounts of lactic acid in the fresh juice samples (< 1.00 g/100 g DM in total DM), whereas other acids were not detected in the analysis.

The ash content of *S. europaea* increased as the cultivation salinity increased due to the high amount of water-soluble salts in the juice fraction. This can be observed when fractions are considered separately: ash content of fibre residue fraction varied between 13.7 and 18.2 g/100 g DM, but the ash content of the juice increased from 49.31 ± 0.19 g/100 g DM (0 mM NaCl) up to 76.02 ± 0.11 g/100 g DM (513 mM NaCl).

#### Tripolium pannonicum

Overall, increased cultivation salinity affected the biomass yield and size of fractions rather than the chemical composition of *T. pannonicum* plants. An inverse relationship was observed between cultivation salinity and the total crude protein content, and the differences between samples were significant (*p* < 0.001). However, considering the green juice and fibre residue fractions from lower salinities (0 mM and 171 mM NaCl) individually, the crude protein content was stable, and changes were non-significant (*p* = 0.259 and *p* = 0.063, respectively). The total lipid content of *T. pannonicum* varied between 1.59 ± 0.46 g/100 g DM (513 mM NaCl) and 2.81 ± 0.04 g/100 g DM (171 mM NaCl). Despite the significant differences in the total lipid content of biomass samples (*p* = 0.002), it was not possible to observe a clear relationship between cultivation salinity and lipid content based on the obtained results. Due to the small amount of biomass available, it was not possible to run the lipid extraction in triplicate for 684 mM NaCl salinity grown biomass. The changes in the lipid content were more pronounced in the fibre residue fraction, as in the juice fractions, the lipid content stayed nearly constant and changes were non-significant (*p* = 0.488).

Significant differences were observed in the total carbohydrate content of samples from different cultivation conditions (*p* < 0.001). As sugars were determined from the extractive-free fraction of the solid samples, only the amount of structural carbohydrates was determined for the samples considered whole (not fractionated). An inverse relationship between cultivation salinity and the content of structural carbohydrates was observed, but only samples cultivated in the two highest salinities were significantly different to the others. Considering the types of sugars separately, the increased cultivation salinity caused significant changes in total glucose and xylose contents of total DM (*p* < 0.001), but there were no significant differences in the arabinose content of biomass (*p* = 0.171). The Klason lignin content of *T. pannonicum* was up to 21.97 ± 1.70 g/100 g DM (171 mM NaCl), and the changes between biomass samples were non-significant (*p* = 0.072).

High concentrations of free sugar monomers were detected from fresh *T. pannonicum* juice, xylose being the most abundant sugar monomer with concentrations of 16.43 ± 0.32 g/100 g DM and 7.99 ± 0.08 g/100 g DM in green juice from 0 mM NaCl and 171 mM NaCl cultivated biomasses, respectively. Fresh green juice was also rich in glucose, but arabinose was not detected in the samples. Only a low amount of lactic acid was measured from fresh juice; other acids were not detected.

**Figure 3:**
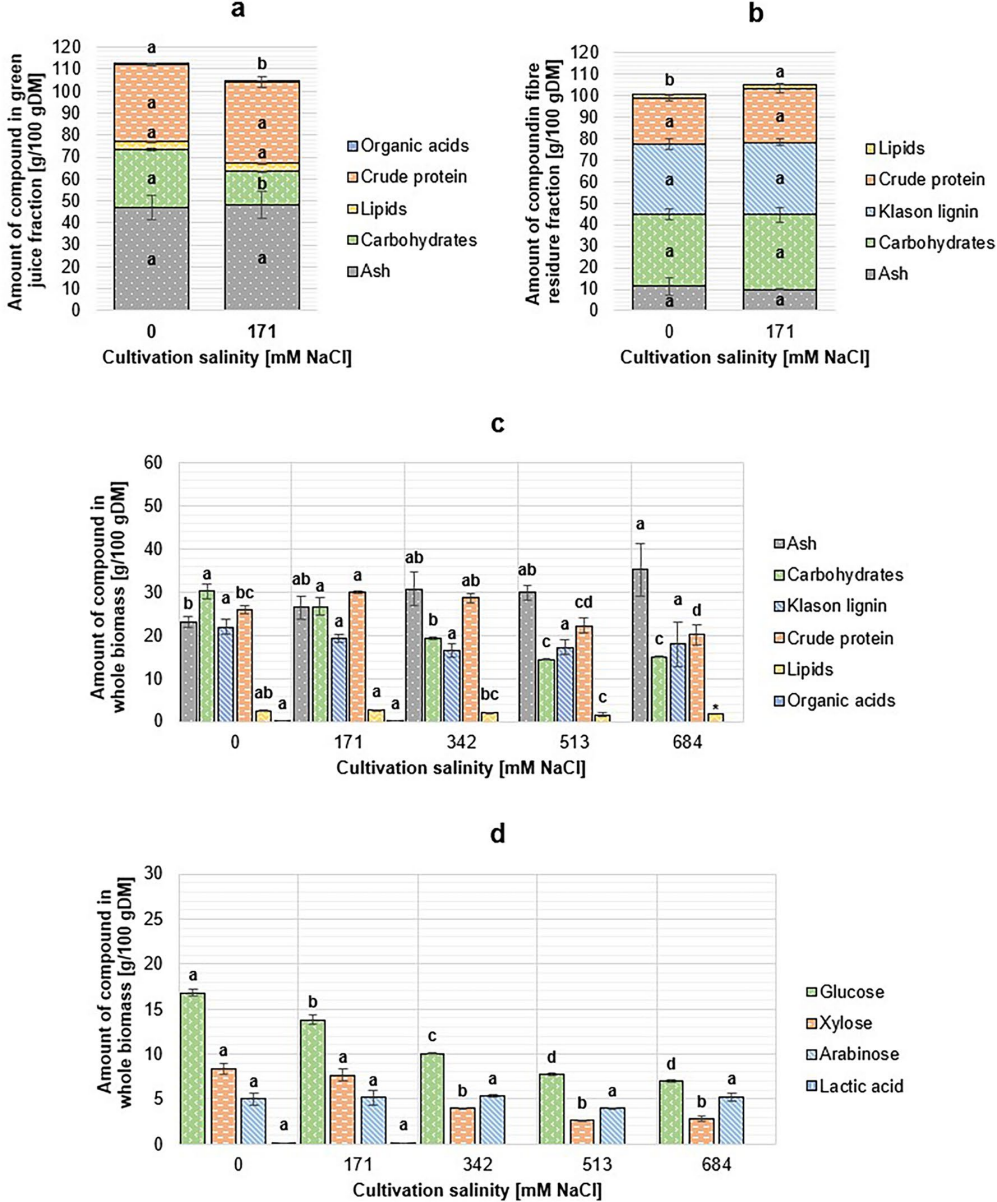
Chemical composition of *Tripolium pannonicum* green juice (**a**), fibre residue (**b**) and whole biomass (**c**), and total sugar profile (**d**). DM: dry matter. For 0 and 171 mM NaCl salinity cultivated samples, the content of a compound in whole biomass was calculated from juice and fibre residue fractions. Only structural carbohydrates were analysed for samples cultivated in 342, 513, and 684 mM NaCl. Biomass samples which were not fractionated were not analysed for their contents of organic acids. Different letters above the bars denote significantly different (*p* < 0.05) results calculated individually for each biochemical group. *Not possible to give the standard deviation for the sample.

The significant difference in the total ash content was only observed in the samples cultivated in the lowest and the highest salinity (*p* = 0.020), whereas changes between other samples were non-significant. When considered separately, the green juice fractions showed no significant change in the ash content (*p* = 0.829). During the composition analysis, the cumulative mass balance of *T. pannonicum* samples exceeded 100%, which is suggested to be caused by the overestimated amount of crude protein and large standard deviations in the ash content results. The composition of *T. pannonicum* biomass fractions are shown in Fig. 3.

#### Crithmum maritimum

**Figure 4:**
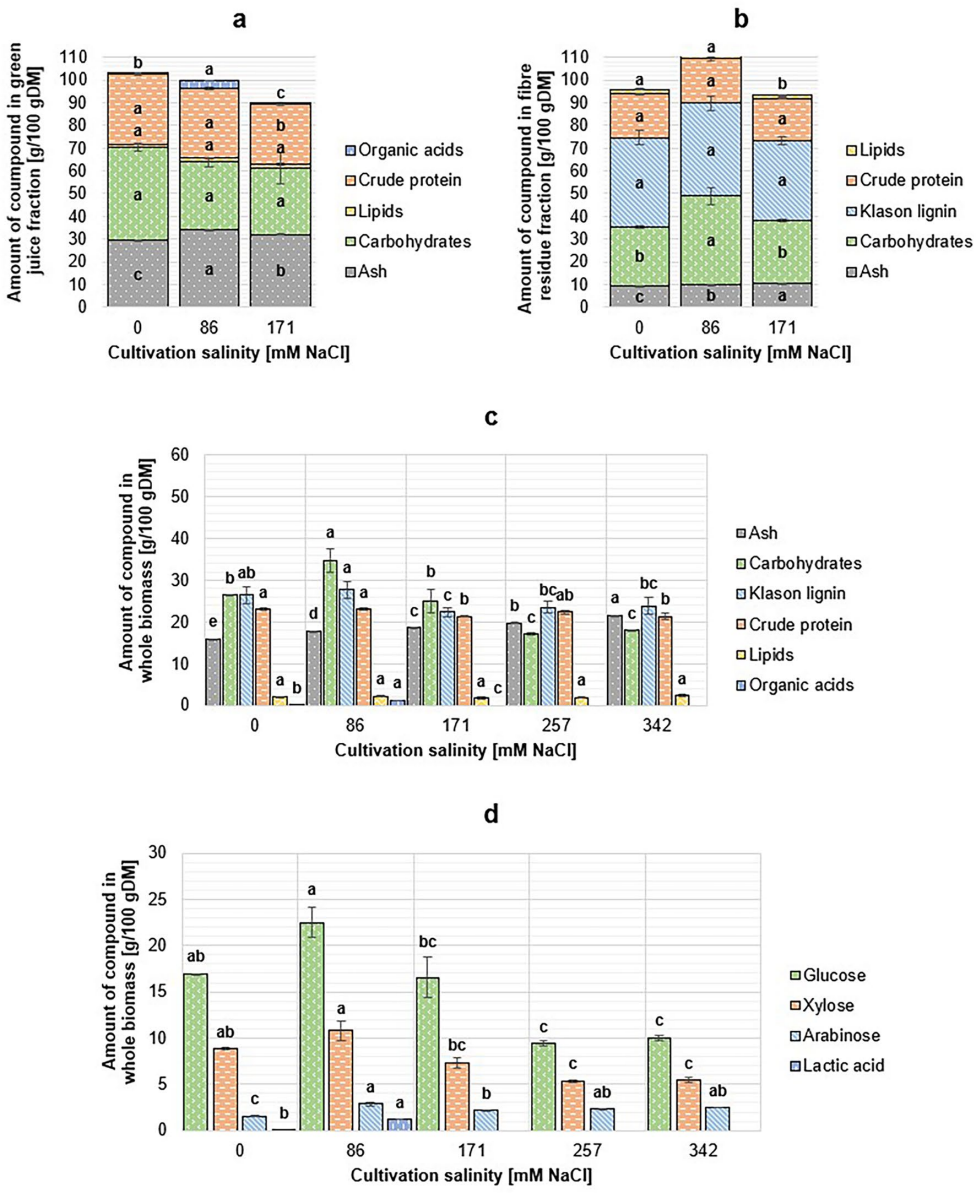
Chemical composition of *Crithmum maritimum* green juice (**a**), fibre residue (**b**) and whole biomass (**c**), and total sugar profile (**d**). DM: dry matter. For 0, 86, and 171 mM NaCl salinity cultivated samples, the content of a compound in whole biomass was calculated from juice and fibre residue fractions. Only structural carbohydrates were analysed for samples cultivated in 257 and 342 mM NaCl. Due to the small concentration of organic acids (< 0.1 g/100 g DM), all bars are not visible in the graphs. Biomass samples which were not fractionated were not analysed for their contents of organic acids. Different letters above the bars denote significantly different (*p* < 0.05) results calculated individually for each biochemical group.

The composition of *C. maritimum* biomass fractions is shown in Fig. 4. Cultivation salinity affected mainly the biomass yield and less the chemical composition of *C. maritimum*. The cumulative mass balance exceeded 100% during the composition analysis of all fibre residue fractions and the juice fraction from biomass grown with 0 mM NaCl. Significant changes (*p* = 0.002) were observed in plants’ total crude protein content, which varied between 21.3 and 23.1 g/100 g DM. When fractions were considered separately, differences were statistically significant in green juice samples (*p* < 0.001) and an inverse relationship between crude protein content and cultivation salinity was observed, whereas no significant differences were observed in the crude protein content of fibre residue fractions (*p* = 0.070). The total lipid content of *C. maritimum* was low (< 2.5 g/100 g DM) in all samples, with changes between biomass batches being non-significant (*p* = 0.045). Most of the total lipids (> 60%) were present in the fibre residue fraction after the screw press. In the green juice fractions, the lipid content was < 2.2 g/100 g DM, and the differences between juice samples were also non-significant (*p* = 0.475).

Significant changes were observed in the total carbohydrate content of the biomass samples (*p* < 0.001). The content of carbohydrates was highest in the plants cultivated in 86 mM NaCl, the total amount of sugars being 34.71 ± 2.86 g/100 g DM. Significant changes were observed in the total contents of glucose (*p* < 0.001), xylose (*p* < 0.001), and arabinose (*p* = 0.002) in total DM of *C. maritimum.* However, when hydrolysed juice samples were considered separately, only non-significant changes were observed in the concentrations of glucose (*p* = 0.510) and xylose (*p* = 0.051), and arabinose was detected only in the sample from 171 mM NaCl cultivated plants in very low concentration (< 0.1 g/100 g DM). Considering the amounts of structural carbohydrates in solid samples, only the pulp fraction from 86 mM NaCl cultivation salinity was significantly different, whereas the carbohydrate content in other solid samples remained nearly constant. Klason lignin content of total DM varied from 22.37 ± 1.09 g/100 g DM (171 mM NaCl) to 27.68 ± 1.99 g/100 g DM (86 mM NaCl) with the *p*-value of 0.004.

Fresh *C. maritimum* juice was rich in free sugar monomers, and concentration varied between samples (*p* < 0.001). In these samples, glucose was the most abundant sugar, and the concentration reached up to 22.14 ± 0.20 g/100 g DM (0 mM NaCl). The amount of xylose was also found to be high, being 18.63 ± 0.47 g/100 g DM in the green juice from plants cultivated with 0 mM NaCl and > 10.00 g/100 g DM in other fresh juice samples. Arabinose was not detected in any of the fresh juice samples. The largest amount of lactic acid was detected from the 86 mM NaCl cultivated sample (3.77 ± 0.00 g/100 g DM), but in other juice samples, lactic acid concentrations were low (< 0. 1 g/100gDM).

Cultivation salinity is directly related to the ash content of the *C. maritimum* biomass (*p* < 0.001).

## Discussion

Halophytes can be divided into two groups: obligate halophytes, which need salt to produce the highest biomass yields, and facultative halophytes, which tolerate salt but have optimal growth in low salinities. In general, dicotyledonous halophyte plants have shown higher salt tolerance compared to monocotyledonous grasses and glass-like species^43^. As an obligate halophyte, *S. europaea* requires salt for optimal growth, and the highest obtained biomass yield from cultivation at 342 mM NaCl salinity (approximately 34 dS/m) aligns with the previous studies reporting the optimal salinity range for *Salicornia* spp. to be 200–400 mM NaCl (corresponding to approximately 20–40 dS/m)^44–47^. In the cultivation study by Araus et al*.*^48^, brackish water (25 dS/m) irrigation lead to taller plants and higher *S. europaea* biomass production compared to seawater (40 dS/m) irrigation. Adaptability to changing environments was shown, as *S. europaea* exhibited sufficient growth in various salinities. In the study by Cárdenas-Pérez et al.^47^ no significant anatomical changes were observed in *S. europaea* cultivated at 200–800 mM salinity, whereas changes in plant cells were observed with extreme salinity treatments (0 mM and 1000 mM NaCl). *S. europaea* exposed to high salinity stress (700 mM) has also exhibited recovery after the stress and as high production of fresh biomass as control plants^49^. *Salicornia* spp. are fully adapted to flooding conditions, which has also been shown to enhance growth^44^.

Unlike *Salicornia* spp., *T. pannonicum* and *C. maritimum* are considered facultative halophytes. Uno et al.^50^ and Ueda et al.^51^ have reported *T. pannonicum* growth inhibition in salinities above 300 mM NaCl. Obtained results are aligned with these observations, as biomass yield was significantly lower when salinity increased to 342 mM NaCl. Wiszniewska et al*.*^52^ also obtained the optimal *T. pannonicum* production at non-saline conditions but reported a non-significant difference in yields in 150 mM and 300 mM NaCl cultivated plants. Turcios et al.^22^ reported *T. pannonicum* being able to withstand salinities up to 770 mM (45 g/l NaCl). Even if *T. pannonicum* survived under 684 mM NaCl salinity, the biomass yield was very low, and only small changes in the chemical composition of the biomass were observed. Al-Hawija et al*.*^53^ also reported *T. pannonicum* seeds being able to germinate under salinity up to 600 mM, but the germination percentage decreased from 78 to 24% compared to non-saline conditions. Considering obtained results and previous literature, *C. maritimum* tolerates lower NaCl concentrations than the other two species. This can be explained by the type and the natural habitat of the species. According to eHALOPH database^54^, *S. europaea* and *T. pannonicum* are classified as hydrohalophytes found in salt marshes, which can be affected by tidal changes in coastal areas, whereas *C. maritimum* is a chasmophyte found in rocky seashores. In addition, plants have several mechanisms to continue their growth and development under salt stress conditions, including the restriction of Na^+^ uptake and exclusion, cellular compartmentalisation of Na^+^ in the vacuole, antioxidant regulation, compatible solutes (osmolytes), morphological adaptations, among others. Germination strategies of halophyte seeds also show high variability depending on plant population and their natural habitat^55^. The mechanism used depends on the group of plants, glycophytes or halophytes, and on each species, resulting in a wide range of tolerance to salinity. For example, Ben Amor et al*.*^56^ and Ben Hamed et al.^57^ reported a significant reduction in *C. maritimum* yields in salinities higher than 200 mM NaCl and at 300 mM NaCl, respectively, but showed enhanced or unchanged growth under moderate 50 mM NaCl and 100 mM NaCl salinities, respectively. Similarly, Martins-Noguerol et al.^58^ defined the optimal cultivation salinity for *C. maritimum* in greenhouse conditions to be 50 mM NaCl (approximately 5 dS/m). Regardless of the lower total biomass production and longer cultivation time, the utilisation of *C. maritimum* could be feasible, as the plant has been reported to be rich in valuable bioactive compounds^35,36^.

It must be taken into consideration that the cultivation of plants in different growth media (hydroponic system or cultivation in soil) can also lead to different plant growth responses and biochemical compositions. However, the use of hydroponic cultivation has increased in importance worldwide due to the known advantages, mainly in the efficient use of the resources, being necessary to carry out research in this field. In addition, under hydroponic conditions and for research purposes, the concentrations of salt and nutrients can be easily and precisely controlled, allowing an accurate comparison between the different treatments. The cultivation system to select also depends on other factors. Different cultivation systems provide varying capabilities to control the salinity^7^, which among other things, such as targeted products, has to be taken into account when choosing the cultivation practices and following processing methods. As *S. europaea* is an annual plant, it could provide an interesting species for crop rotation, where it would be used in the remediation process and to uptake the excessive salt from the substrate, which may inhibit the growth of other crops.

This study presents a broad overview of the composition of green fractionated halophyte biomasses grown in the hydroponic system, providing information for planning potential biorefinery processes. The crude protein content of studied halophytes was relatively high, and these species could have the potential for protein production. Results for total crude protein content for *S. europaea* were aligned with amounts previously reported for *Salicornia* spp.^19,26^. The content of soluble protein in *S. europaea* has previously shown to be relatively stable and content to decrease only when exposed to very high salinities^47^. The crude protein content was lower in *S. europaea* samples cultivated in more optimal conditions regarding biomass yield; thus, the actual crude protein content was 12.7% higher in plants cultivated in 171 mM NaCl than plants cultivated in 342 mM NaCl. Therefore, the cultivation in 171 mM NaCl could be more desirable for a biorefinery targeting maximum protein production when assuming that all crude protein could be extracted as true protein and changes in the biomass yields would be significant. Even if the total crude protein content is nearly the same in 171 mM NaCl and 684 mM NaCl cultivated samples, due to significantly lower biomass yield obtained with 684 mM, the amount of total protein would decrease. Therefore this cultivation condition cannot be suggested. For *T. pannonicum*, the crude protein content was higher than previously reported for species^22^ and six other species in the Asteraceae family^59^. The crude protein content of *T. pannonicum* DM was comparable to widely used legumes, such as chickpea (24.0 g/100 g), lentil (26.1 g/100 g) or green pea (24.9 g/100 g)^60^, which makes it the most interesting species for protein production. However, the presence of non-protein free amino acids (e.g. asparagine) may cause an overestimation in protein content^61^, as well as high content of nitrate, which depending on cultivation practices, may also become an anti-nutritional factor in *T. pannonicum*^4^. Nitrogen is also present in chlorophylls, and high concentration may affect the estimation of protein content. Therefore, an amino acid analysis would be needed to carry out in further investigations. Also, *C. maritimum* showed relatively high crude protein content, and previous studies have shown optimal cultivation conditions to increase the content of essential amino acids in the plant^58^.

Low lipid content was measured from all studied plant species, which is typical for succulent halophytes^62^. For *T. pannonicum*, the total lipid content was lower than values previously reported in the literature^22^. Abiotic stresses, such as high or low salinity, could increase the antioxidant capacity and production of certain protective non-polar compounds, such as carotenoids, in plants^26,47,63^. Therefore, analysis of fatty acid profile and characterisation of other non-polar compounds (e.g. pigments and tocopherols) from lipid-enriched fractions could be desired to evaluate the feasibility of lipid separations as part of the biorefinery process.

With respect to total biomass yield, optimal cultivation conditions seem to increase the lignocellulosic fraction in the *S. europaea* biomass. This can be linked to the larger plant size obtained from the cultivation under optimal salinity^47,48^. Therefore, biomass cultivated in these conditions could be more suitable for biorefinery targeting cellulose and hemicellulose derivatives, cellulose being present mainly in the lignified stems^10^. Regardless of the significantly higher carbohydrate content in *C. maritimum* from 86 mM NaCl salinity cultivation, the actual amount of carbohydrates in obtained fresh biomass was still higher in plants cultivated in 0 mM NaCl due to higher biomass yield. Klason lignin content of *S. europaea* was found to be low, and it is aligned with acid-insoluble lignin contents previously reported for *Salicornia* species^18,19,64^. Low lignin content may indicate biomass to be non-recalcitrant, allowing less severe processing conditions, especially after the removal of extractive material. In *C. maritimum* and *T. pannonicum* biomass, Klason lignin content was higher than *S. europaea*, and the insoluble lignin content was aligned with 18.2 g/100 g DM previously reported for *T. pannonicum*^22^. Studied facultative halophytes had a high concentration of available sugars in the juice, making them interesting for processes utilising micro-organisms. Compared to forage alfalfa juice, which has been suggested as media for lactic acid fermentation^65^, the amount of available glucose is similar in *T. pannonicum* and nearly triple in *C. maritimum* cultivated in moderate salinities. The high amount of available xylose could also make *T. pannonicum* a potential feedstock for the production of pentose-derived platform chemicals.

Halophytes are known to accumulate salt and other minerals in their tissues, and high ash contents were also measured from studied biomass. Ushakova et al.^66^ showed increased sodium intake and decreased potassium, calcium, and magnesium uptake of *S. europaea*, which aligns with obtained results. Studied halophytes, especially *C. maritimum* and *S. europaea,* were rich in calcium (see Supplementary Table S1 and Supplementary Table S3), which has been shown to have an essential protective role in the salt tolerance of plants growing in saline conditions^67^. Compared to the other studied species cultivated in the same salinities, *C. maritimum* exhibited lower salt accumulation.

## Conclusion

*S. europaea*, *T. pannonicum,* and *C. maritimum* were cultivated in different salinity conditions, and *S. europaea* yielded the most biomass in 342 mM NaCl (approximately 34 dS/m) salinity, whereas facultative halophyte species exhibited the highest biomass production in non-saline conditions. *T. pannonicum*, and especially *S. europaea*, could be potential crops due to their higher biomass yields and shorter cultivation time. Obtained biomass was fractionated to green juice and fibre residue, and the chemical composition of the fractions were analysed. Significant differences were observed between biomass batches cultivated under different salinities, and this study is the first one to report the composition of the species after green fractionation. All species exhibited high crude protein content. Therefore, they can be seen as potential feedstocks for biorefinery targeting green protein production. Obtained results can be used to plan possible processing routes for halophyte-based biorefinery. Still, halophytes and their suitability for different applications should be further explored as a part of the green transition and development of marginal lands.

## Supplementary Information

Supplementary Tables.

## Data Availability

The data generated and analysed during the study is available from the corresponding author on request.
