# Supplementary material for: Cultivation and characterisation of Salicornia europaea, Tripolium pannonicum and Crithmum maritimum biomass for green biorefinery applications
Source: Sci Rep. 2022 Nov 28;12:20507. doi: 10.1038/s41598-022-24865-4 (PMC9705282; doi:10.1038/s41598-022-24865-4)
Supplement: Supplementary file 1 — Supplementary Tables. [file 41598_2022_24865_MOESM1_ESM.pdf]

Cultivation and Characterisation of *Salicornia europaea*, *Tripolium pannonicum* and *Crithmum maritimum* Biomass for Green Biorefinery Applications

Laura S. S. Hulkko[1*], Ariel Turcios[2], Stéphane Kohnen[3], Tanmay Chaturvedi[1], Jutta Papenbrock[2], Mette Hedegaard Thomsen[1]

[1] AAU Energy, Aalborg University, Niels Bohrs Vej 8 6700 Esbjerg, Denmark
[2] Institute of Botany, Leibniz Universität Hannover, Herrenhäuser Straße 2 30419 Hannover, Germany
[3] CELABOR, Avenue du Parc n°38 4650 Chaineux, Belgium
[*]Corresponding author: lssh@energy.aau.dk

Supplementary Information

Mineral Analysis of Halophyte Biomasses

Salicornia europaea

Results of the mineral analysis were calculated on the basis of fresh weight (FW). Higher cultivation salinity increased the sodium content of *S. europaea* biomass and decreased the concentration of other minerals. Minerals in the ash fractions of *S. europaea* are presented in **Supplementary Table S1**. Aluminium was detected from the ash fraction of *S. europaea* juice, but as biomass was cultivated in a hydroponic system without contamination, a plausible source is the foil dishes in which the juice was dried.

Supplementary Table S1 Minerals present in the ash fraction of *Salicornia europaea* biomass cultivated under different salinities. The amount of compound in total ash was calculated from juice and fibre residue ash fractions. n.d.: amounts > 100 ppm not detected.

Element [mg/100 gFW]	0 mM NaCl	171 mM NaCl	342 mM NaCl	513 mM NaCl	684 mM NaCl
**Aluminium**	0.48	3.26	< 2.94	< 4.32	5.01
**Barium**	0.11	< 0.03	n.d.	n.d.	n.d.
**Calcium**	219.45	46.30	28.26	21.31	24.48
**Copper**	< 0.07	< 0.09	< 0.09	< 0.10	< 0.09
**Iron**	0.34	0.37	0.29	0.23	0.31
**Potassium**	478.05	238.18	158.29	115.04	112.38
**Magnesium**	49.47	30.87	15.85	10.12	8.87
**Manganese**	0.15	0.09	0.07	0.09	0.16
**Molybdenum**	n.d.	n.d.	n.d.	n.d.	n.d.
**Sodium**	494.12	1,191.11	1,328.44	1,463.83	1,492.54
**Phosphorus**	30.93	22.48	19.33	18.23	16.73
**Rubidium**	0.03	n.d.	n.d.	n.d.	n.d.
**Strontium**	0.27	0.06	< 0.04	n.d.	n.d.
**Vanadium**	0.03	n.d.	n.d.	n.d.	n.d.
**Zinc**	0.20	0.13	0.09	0.08	0.11

Tripolium pannonicum

For *T. pannonicum*, the mineral analysis was performed using the ash fractions of whole biomass samples. As the biomass yields from high salinity conditions were low, it was not possible to run the mineral analysis for these samples due to a lack of plant material. Besides the increase in the sodium concentration, the higher cultivation salinity also increased the amount of phosphorus in biomass. The amounts of most minerals in *T.*

pannonicum ash decreased from 0 mM NaCl to 171 mM NaCl cultivation salinity and got higher again when the cultivation salinity increased to 342 mM NaCl. However, due to a lack of replicates, it was not possible to evaluate the significance of these changes. Minerals in the ash fractions of *T. pannonicum* are presented in **Supplementary Table S2.**

Supplementary Table S2 Minerals present in the ash fraction of *Tripolium pannonicum* biomass cultivated under different salinities. Not enough material was available to perform mineral analysis for biomass cultivated in high salinities. n.d.: amounts > 100 ppm not detected.

Element [mg/100 gFW]	0 mM NaCl	171 mM NaCl	342 mM NaCl	513 mM NaCl	684 mM NaCl
**Aluminium**	0.08	0.07	0.09	n/a	n/a
**Barium**	0.06	0.03	0.04	n/a	n/a
**Calcium**	112.17	54.76	62.62	n/a	n/a
**Copper**	0.08	0.07	0.11	n/a	n/a
**Iron**	0.35	0.27	0.49	n/a	n/a
**Potassium**	743.29	496.30	506.00	n/a	n/a
**Magnesium**	31.65	10.38	12.03	n/a	n/a
**Manganese**	0.16	0.13	0.32	n/a	n/a
**Molybdenum**	0.02	0.02	0.06	n/a	n/a
**Sodium**	145.22	502.85	1,078.87	n/a	n/a
**Phosphorus**	52.15	54.90	77.52	n/a	n/a
**Rubidium**	0.04	0.03	n.d.	n/a	n/a
**Strontium**	0.11	0.05	0.06	n/a	n/a
**Vanadium**	n.d.	n.d.	n.d.	n/a	n/a
**Zinc**	0.25	0.16	0.35	n/a	n/a

Crithmum maritimum

The mineral analysis showed that increased cultivation salinity decreased the uptake of calcium, potassium and magnesium, whereas the concentrations of phosphorus and iron increased with cultivation salinity. Minerals in the ash fractions of *C. maritimum* are presented in **Supplementary Table S3.**

Supplementary Table S3 Minerals present in the ash fraction of *Crithmum maritimum* biomass cultivated under different salinities. n.d.: amounts > 100 ppm not detected.

Element [mg/100 gFW]	0 mM NaCl	86 mM NaCl	171 mM NaCl	257 mM NaCl	342 mM NaCl
**Aluminium**	0.37	0.40	0.62	0.47	0.69
**Barium**	0.13	0.11	0.11	0.08	0.07
**Calcium**	228.81	179.08	152.16	139.42	115.42
**Copper**	0.06	0.07	0.07	0.06	0.05
**Iron**	0.37	0.35	0.45	0.48	0.52
**Potassium**	582.93	515.19	434.36	279.37	220.58
**Magnesium**	14.51	11.76	12.33	9.57	8.99
**Manganese**	0.20	0.18	0.20	0.20	0.12
**Molybdenum**	0.02	0.02	0.02	0.02	0.03
**Sodium**	11.47	193.25	352.43	555.99	816.29
**Phosphorus**	82.93	96.32	98.86	116.89	106.72
**Rubidium**	0.03	0.03	0.03	0.02	0.02
**Strontium**	0.20	0.15	0.15	0.11	0.10

**Vanadium**	n.d.	n.d.	n.d.	n.d.	n.d.
**Zinc**	0.11	0.13	0.20	0.23	0.18

In all species, the sodium content increased drastically when moving from non-saline conditions to 171 mM NaCl cultivated samples, and per mass unit of fresh biomass, the sodium concentration increased 340.96 mg/g FW in *C. maritimum*, 357.63 mg/g FW in *T. pannonicum*, and 696.99 mg/g FW in *S. europaea*. In the 342 mM NaCl salinity, sodium concentration per mass unit of fresh biomass further increased 463.86 mg/g FW, 576.0 mg/g FW, and 137.33 mg/g FW in *C. maritimum*, *T. pannonicum*, and *S. europaea*, respectively.
